# A new experimental community pharmacy internship module for undergraduate pharmacy students in western Nepal: overview and reflections

**DOI:** 10.3352/jeehp.2017.14.18

**Published:** 2017-08-16

**Authors:** Sangita Timsina, Bhuvan K.C., Dristi Adhikari, Alian A. Alrasheedy, Mohamed Izham Mohamed Ibrahim, Atisammodavardhana Kaundinnyayana

**Affiliations:** 1 School of Health and Allied Sciences, Pokhara University, Kaski, Nepal; 2 School of Pharmacy, Monash University Malaysia, Subang Jaya, Malaysia; 3 Unaizah College of Pharmacy, Qassim University, Qassim, Saudi Arabia; 4 College of Pharmacy, Qatar University, Doha, Qatar; Hallym University, Korea

**Keywords:** Community pharmacies, Internship, Pharmacy education, Nepal

## Abstract

Community pharmacies in Nepal and other South Asian countries are in a mediocre state due to poor regulation and the fact that many pharmacies are run by people with insufficient training in dispensing. This has led to the inappropriate use of medicines. The problems due to poor regulation and the mediocre state of community pharmacies in South Asia encompass both academia and clinical practice. In this paper, a 2-week community pharmacy internship programme completed by 2 graduating pharmacy students of Pokhara University (a Nepalese public university) at Sankalpa Pharmacy, Pokhara, Nepal is illustrated. During the internship, they were systematically trained on store management, pharmaceutical care, counselling skills, the use of medical devices, pharmaceutical business plans, medicine information sources, and adverse drug reaction reporting. An orientation, observations and hands-on training, case presentation, discussion, and feedback from 2 senior pharmacists were used as the training method. A proper community pharmacy internship format, good pharmacy practice standards, and a better work environment for pharmacists may improve the quality of community pharmacies.

## Introduction

Nepal is a low-income country located in South Asia, between India and China [1]. It has an area of 147,181 square kilometres and a population of approximately 28.5 million in 2015 [1,2]. The state of public services such as education and healthcare is poor, as is access to resources such as roads, electricity, and water [1,2]. Vital health indicators are likewise poor; in particular, the maternal mortality rate (per 100,000 live births) is 170 (2013) and the under-5 mortality (per 1000 live births) is 53 (2011) [2]. Moreover, healthcare spending per capita in 2014 was 39.86 US dollar [2]. These healthcare indicators show that Nepal has much to do to improve the overall state of healthcare.

Pokhara University, a public university established in 1997, started an undergraduate pharmacy program (bachelor of pharmaceutical sciences) in 2001 [3]. The program is a 4-year undergraduate degree with education, practice, and internship components. After completing the undergraduate pharmacy degree, one has to appear for a registration examination to be registered as a pharmacist with the Nepal Pharmacy Council [4]. The curriculum of the undergraduate pharmacy degree of Pokhara University comprises basic sciences, applied pharmaceutical sciences, and pharmacy practice [3]. The pharmacy practice component covers both theoretical and practical aspects of pharmacy practice. It includes both institutional pharmacy practice (i.e., hospital and clinical pharmacy) and practice in community-based settings (i.e., community pharmacy). As part of the community pharmacy course, the students have to complete a mandatory 2-week community pharmacy internship [3].

The Sankalpa Foundation is a not-for-profit organization that works for holistic health development. It is composed of 4 units: a holistic health unit, a consultancy unit, a community pharmacy unit, and an advocacy and outcome research unit [5]. The Sankalpa Pharmacy is a community pharmacy under the umbrella of the Sankalpa Foundation, located in Pokhara, Nepal. The Sankalpa Foundation has conducted different projects, such as an over-the-counter medicine safety project, patient education project, and pharmacist-led diabetes and hypertension management projects that are run through the Sankalpa Pharmacy [5]. The Sankalpa Pharmacy also provides drug information services to patients and other clients at the community level, and has provisions for adverse drug reaction reporting and documentation [5].

Two final-year students of Pokhara University were posted in the Sankalpa Pharmacy for their internship under the supervision of a senior pharmacist (a pharmacist with a postgraduate degree in pharmacy, registered with the Nepal Pharmacy Council and more than 5 years’ experience in both academia and community pharmacy practice). Thus, the objective of this paper was to illustrate the 2-week community pharmacy internship programme completed by 2 graduating pharmacy students of Pokhara University (a Nepalese public university) at Sankalpa Pharmacy, Pokhara, Nepal.

The pharmacist team of the Sankalpa Foundation designed the content of the internship. The training objectives and learning outcomes were discussed and reviewed following a discussion session with 10 final-year undergraduate pharmacy students. The community pharmacy training format and this write-up were reviewed and approved by the institutional review board of the Sankalpa Foundation. Table 1 summarizes the internship module.

## Text

### Teaching and learning activities

The community pharmacy internship programme, which is integrated in the fourth academic year, aims to expose students to health services and the delivery thereof at the community level. Observations of the functioning of a community pharmacy and the interaction of interns with health service users help the students to develop a deeper knowledge of the health services and medicines provided by community pharmacies [3]. It also allows the students to learn about the functioning of community pharmacies and the nature of interactions between health service users and community pharmacists [3].

The students (both students together) were posted in the pharmacy from 8 AM to 6 PM with two 30-minute refreshment breaks. The students were posted in the pharmacy outlet unit, pharmacy store unit, and pharmacy office where the documentation and pharmacy services were provided. Each day’s session begun with a briefing on the learning objective, following which students were allowed to go to their respective unit and carry out their assigned activities. At the end of each day, a discussion and question-and-answer session were carried out. The internship programme included an orientation session, an observation and hands-on training session, a case presentation session, a discussion session, a presentation by students, and an active discussion session with 2 senior pharmacists. At the end of the entire session, a roundtable discussion and feedback session were carried out. After completing the internship, students prepared and submitted a report to the Pokhara University and Sankalpa Pharmacy.

### Reflections

In this community pharmacy internship, the students received an opportunity to learn about various pharmacy services and to be trained in various community pharmacy-related skills. They received hands-on training on blood pressure and blood glucose measurements, body weight measurements, and measuring the respiratory rate and body temperature. They were also trained on the use of devices such as nebulizers, pressurized meter-dose inhalers, dry-powder inhalers, and other medication aid devices. Furthermore, they also observed the logistics and management process of medicines, pharmacist-managed medication management programmes, and drug information including adverse drug reaction provisions and operations. However, the students had to complete the entire internship module in 2 weeks, and the time limit may have affected their learning. Given the number of different topics and the range of clinical skills, observational sessions, hands-on training, and feedback/discussion sessions covered in this internship, students would benefit from a longer duration of the internship (3 to 4 weeks).

“Posting at Sankalpa Pharmacy as a community pharmacy practice trainee was truly a learning experience, where I acquired different skills related to pharmacy practice. It has provided me with both insights and practical skills related to running a community pharmacy. The exposure to different activities within a community pharmacy has improved my performance as a community pharmacist as well as health care provider.” Intern #ab2.

### Challenges of community pharmacy practice in the South Asian context

The community pharmacy internship format is much more rigorous and covers a longer period in countries that have a well-established pharmacy practice, especially in the developed Organization of Economic Cooperation and Development countries [6]. However, the overall pharmacy sector is still in its evolving phase in Nepal and much of South Asia, meaning that the community pharmacy sector is not well established. Most pharmacies are run as a drug shop or a retail shop, most often by a person with only basic training in drug dispensing [7,8]. This has negatively affected the outlook of the pharmacy profession. Community pharmacies are seen as sales-oriented drug sellers, not as pharmacy service providers. Thus, the community pharmacy sector currently faces multifactorial problems in the South Asian context.

The socioeconomic and sociopolitical circumstances of South Asian countries have affected the development and delivery of healthcare services. For example, appropriate legislative reforms and regulatory institutions to ensure quality pharmacy services are lacking in Pakistan, which affects the functioning of community pharmacies in the country [9]. Likewise, the quality of medicine use in Bangladesh has been negatively affected by private drug shops, as many of them are unregulated and managed by non-pharmacists (persons who learn to dispense through an apprenticeship), and the regulatory processes governing them are complicated [10]. The overall healthcare spending by government is inadequate in countries such as India, Nepal, Pakistan, and Bangladesh. Furthermore, the regulatory environment is diluted by both the lack of resources and governance problems in much of South Asia. Most community pharmacies do not follow good pharmacy practice guidelines and are sales- and product-oriented, instead of focusing on pharmacy services [8,11]. Pharmacists and pharmacy assistants are often unable to enter the community pharmacy sector due to a lack of jobs in the field and the inability of a new community pharmacy to penetrate the existing market.

On the academic front, the curriculum of pharmacy schools that train pharmacists and pharmacy assistants do not cover the theory and practice of community pharmacy well. For example, in much of South Asia, pharmacy students at both the undergraduate and diploma level do not undergo enough practical training on managing symptoms in pharmacy, extemporaneous preparation, and simulated patient counselling sessions, and the educational programme lacks a format that guides students’ learning during community pharmacy internships [3,11].

Finally, the healthcare system is still in its evolving phase in South Asia in comparison to developed Western countries in terms of healthcare financing, health policy, management, and delivery. Likewise, the social perception and recognition of pharmacists is very low, especially in the community pharmacy sector, due to the business- and sales-oriented outlook of the sector [12]. Thus, pharmacists and pharmacy assistants are very reluctant to join the community pharmacy sector, as they do not see a viable future in the area.

### Replicability of the internship module in the South Asian context

To improve the standard of practice of community pharmacy in South Asia, the pharmacy sector must work through the existing healthcare system and gradually improve existing retail pharmacies. Pharmacy stakeholders, such as pharmacy councils, pharmacy schools, and pharmacy professional associations, should join hands with government and retailers’ associations and develop a mechanism through which pharmacy students can complete community pharmacy internships in retail pharmacies. This will create an environment that facilitates mutual appreciation, in which students can learn about the existing circumstances of retail pharmacy and try to practice their community pharmacy knowledge and skills. Thus, pharmacy schools should develop a simple format that provides learning goals and activities that students can do during the internship. We believe that the proposed experimental community pharmacy internship module may be used as a basis for the development of training modules in South Asian countries and other countries that do not have well-defined modules.

### Limitation

Only 2 pharmacy students were involved in the community pharmacy internship. The small number of internship students is a limitation of this study, as it affected the feedback and reflections provided by the participants.

## Conclusion

The community pharmacy sector can play a huge role in promoting the safe and effective use of medicines in Nepal and elsewhere in South Asia. This internship programme shows that a good community pharmacy placement contributes to students’ community pharmacy practice skills and to community pharmacy practice standards. However, the business motive of community pharmacies (instead of focusing on providing pharmacy services), unprofessional outlook, lack of social recognition, inadequate training in community pharmacy in the undergraduate curriculum, and the inadequate financial rewards and financial viability of community pharmacy hinder the improvement of the community pharmacy sector in South Asia. Thus, a concerted effort from all health stakeholders is required to improve the standard of practice of community pharmacies in South Asia.

## Figures and Tables

**Table 1.** Activity schedule of the 2-week internship programme

Days	Activities	Detail
Day 1	Orientation	Briefing on pharmacy operation and services
		Discussion about the objective of the session
		Daily work schedule, including learning clinical skills and responding to symptoms
Day 2	Observation	Pharmacy layout
		Arrangement of drugs in pharmacy (outlet and store)
		Types of medicines available
		Requirements for medication storage
Day 3	Store management	Storage of drugs according to their volume of use
		First-in/first-out method
Day 4 and day 5	Pharmaceutical care for the community	Management of chronic and long-term health problems though pharmacy
		Responding to symptoms in community pharmacy
		Dealing with special health issues
		Proper information about family planning methods
		Health screening
Day 6	Counselling skills	Basic counselling skills
		Counselling for stigmatized patients
		Use of pharmacological and non-pharmacological approaches during counselling
Day 7	Practical skills	Use of medical devices
		Applying methods of physical examination
Day 8	Business plan	Drug selection criteria and drug procurement activities
		Inventory management
		Break-even point analysis
		Legal procedure for establishing a community pharmacy
Day 9	Waste management	Handling of damaged and expired drugs
		Disposal of wastage
Day 10	Medicine information source	Use of available information sources in pharmacy
		Development of informational material by the pharmacy such as leaflets, booklets, and other materials for educational purposes
Day 11	ADR reporting and pharmacovigilance	Determination of ADR by various assessment scales
		Reporting of ADR to regional pharmacovigilance center
Day 12	Future prospects	Potential for community pharmacy-managed services in the Nepalese context

ADR, adverse drug reaction.

## Supplementary materials

Supplement 1. Audio recording of the abstract.
